# The Synergy and Mode of Action of* Cyperus rotundus* L. Extract Plus Ampicillin against Ampicillin-Resistant* Staphylococcus aureus*

**DOI:** 10.1155/2018/3438453

**Published:** 2018-04-03

**Authors:** Prairadda Cheypratub, Wilairat Leeanansaksiri, Griangsak Eumkeb

**Affiliations:** School of Preclinic, Institute of Science, Suranaree University of Technology, Nakhon Ratchasima, Thailand

## Abstract

*Cyperus rotundus *L. has been used for pharmaceutical applications including antibacterial infections. Nevertheless, there is still no data regarding the mode of actions. This study aimed to determine the antibacterial activity and mode of actions of* Cyperus rotundus* extract (CRE) against ampicillin-resistant* Staphylococcus aureus* (ARSA) which poses a serious problem for hospitalized patients. The majority of chemical compounds of CRE were flavonoids and alkaloids. The minimum inhibitory concentrations (MICs) for ampicillin and CRE against all ARSA strains were 64 *μ*g/ml and 0.5 mg/ml, respectively. Checkerboard assay revealed synergistic activity in the combination of ampicillin and CRE at the lowest fractional inhibitory concentration index (FICI) at 0.27. The killing curve assay had confirmed the synergistic and bactericidal activity of the combination against ARSA. Electron microscopic results showed that these ARSA cells treated with this combination caused peptidoglycan and cytoplasmic membrane (CM) damage and average cell areas significantly smaller than control. Also, this combination caused an increase in CM permeability of ARSA. CRE revealed the inhibitory activity against *β*-lactamase. It is normally known that some drugs are derived from flavonoids or alkaloids. So, this CRE proposes the potential to develop a novel adjunct phytopharmaceutical to ampicillin for the remedy of ARSA.

## 1. Introduction

Antibiotic resistance has become a serious problem threatening to public health and medical implications. Many hospital-acquired infections are caused by multidrug-resistant microorganisms such as methicillin-resistant* Staphylococcus aureus* (MRSA) which has infiltrated the community level. Recent studies demonstrated that *β*-lactamase inhibitors with *β*-lactam structures caused the induction of *β*-lactamase expression and hyperproduction of *β*-lactamase [1–3]. Therefore, these currently most available *β*-lactamase inhibitors can also lose their activities through the same mechanism as the *β*-lactam antibiotics [3]. These reasons indicate that the other alternative *β*-lactamase inhibitors with non-*β*-lactam structures are urgently needed in order to overcome the *β*-lactam antibiotic resistance. The* Cyperus rotundus *L.* (C. rotundus)*, known as nutgrass or motha, was widely used in several fields of pharmacological applications including antibacterial infections. In addition,* Cyperus rotundus* crude extract (CRE) contains bioactive compounds such as alkaloids, flavonoids, and polyphenols [4–8] which may be responsible for MRSA inhibition. Some of the flavonoid compounds, such as galangin, quercetin, and baicalein, with non-*β*-lactam structure, can act as a *β*-lactamase inhibitor [3]. Thus, this study aimed to investigate the bioactive compounds present in CRE and to test the antibacterial activity and synergism with ampicillin of CRE against ARSA. Some elementary mode of actions, such as a cytoplasmic membrane (CM) permeabilization, enzyme assay, and transmission electron microscopy, were also studied.

## 2. Materials and Methods 

### 2.1. Medicinal Plant and Bacterial Strains

*C. rotundus* tubers and rhizomes were purchased from a local herb market in Nakhon Ratchasima, Thailand. Plant specimens (voucher specimen BKF No. 194436) were authenticated and deposited at Forest Herbarium, Bangkok, Thailand.

Clinical isolates of* Staphylococcus aureus *ATCC 29213 and ampicillin-resistant* Staphylococcus aureus* DMST 20651 (ARSA), 20652, and 20653 were obtained from the American Type Culture Collection (ATCC), the USA, and Department of Medical Science, National Institute of Health, Ministry of Public Health, Thailand, respectively.

### 2.2. Chemicals and Media

Ampicillin, nisin, and *β*-lactamase type IV from* Enterobacter cloacae *were obtained from Sigma-Aldrich. Cation-adjusted Mueller-Hinton broth (CAMHB) and Mueller-Hinton agar (MHA) were obtained from Oxoid (Basingstoke, UK).

### 2.3. Preparation of* C. rotundus* Extract

*C. rotundus* tubers and rhizomes were completely dried and grounded into a fine powder. The powder was extracted by 95% ethanolic soxhlet extraction at 70°C for 8 h [4] followed by filtration through Whatman No. 1 filter paper. Evaporation was operated under reduced pressure in a rota evaporator at 40°C [4] by slowly reducing the pressure down to 110–70 mbar and freeze-drying under vacuum. The percent yield of CRE was calculated as

(1) $$\begin{array}{c} \%\;\;Yield=\frac{\left[Weight\;\;of\;\;CRE\times 100\right]}{Weight\;\;of\;\;dried\;\;tubers\;\;and\;\;rhizomes}. \end{array}$$

### 2.4. Qualitative Phytochemical Screening

The preliminary qualitative phytochemical screening was preceded for ubiquitous bioactive compounds such as alkaloids, saponins, flavonoids, tannins, glycosides, and polyphenols. The screening test was achieved by using the methods previously described [9–12].

### 2.5. Quantitative Phytochemical Determination

The total alkaloid, flavonoid, and polyphenol contents were determined by liquid chromatography-tandem mass spectrometry (LC-MS/MS) operated with Agilent Technologies 6490 Triple Quad LC/MS coupled with Agilent Technologies 1290 Infinity. The total alkaloid, flavonoid, and polyphenol contents were carried out, with the following setting: Xiao et al. [13] for total alkaloid content and Sanchez-Rabaneda et al. [14] for total flavonoid and polyphenol contents. Rotundine (only the alkaloid found in rhizomes of* C. rotundus* [15, 16]), quercetin, and gallic acid were employed as standard alkaloid, flavonoid, and polyphenol, respectively. The total phytochemical contents were calculated as follows:

(2) Total  alkaloid  content TAC=RE×VmTotal  flavonoid  content TFC=QE×VmTotal  polyphenol  content TPC=GAE×Vm,

where *V* is the volume of extract (ml); *m* is the weight of dry extract (g); RE is a rotundine equivalent (mg/ml); QE is a quercetin equivalent (mg/ml); GAE is gallic acid equivalent (mg/ml).

### 2.6. Agar Disc Diffusion Screening

Agar disc diffusion technique following the method of Ortez [17] and Voravuthikunchai and Kitpipit [18] was applied for primary sensitivity screening test. Briefly, one loopful of* S. aureus* and ARSA strains were separately inoculated in 50 ml of CAMHB for 18 h. Then, the 18 h cultures were adjusted with 0.85% NaCl to achieve 10^8^ cfu/ml by using predetermined bacterial suspension standard curves of absorbance at 500 nm against viable cell count (data not shown) [19]. Next, the bacterial suspensions were thoroughly swabbed on MHA plates. The 10 *μ*l of CRE at 250 mg/ml in dimethyl sulfoxide (DMSO) was dropped onto a sterile paper disc to obtain 2.5 mg/disc. Moreover, 10 *μ*l of 1 mg/ml of ampicillin in distilled water was employed as a positive control and dropped onto a sterile paper disc to acquire 10 *μ*g/disc. The 10 *μ*l of 10% DMSO was prepared as a negative control. All discs were put on the overdried surface of MHA plates and were incubated at 37°C for 18 h. Finally, diameters of inhibition zone were measured and compared to positive and negative controls.

### 2.7. Minimum Inhibitory Concentration (MIC) Determination

MIC by agar dilution technique was performed in accordance with previous reports and some modifications [20, 21]. Briefly, the 18 h of bacterial cultures were tested on MHA containing various concentrations of CRE and ampicillin. The twofold serial dilution stock of CRE in DMSO was started from 320 to 0.625 mg/ml while the twofold serial dilution stock of ampicillin in distilled water was started from 10240 to 0.0195312 *μ*g/ml. The 0.2 ml of each twofold serial dilution stock was added in 1.8 ml of melted MHA in sterile 6-well plates and then mixed thoroughly and allowed them to solidify. This made the final concentrations of CRE in MHA range from 0.0625 to 32 mg/ml when the final concentrations of ampicillin in the MHA range from 0.00195312 to 1024 *μ*g/ml. The 0.2 ml of DMSO without CRE in 1.8 ml MHA and 0.2 ml of distilled water without ampicillin in 1.8 ml MHA were subjected to positive controls for the use of the CRE and antibiotic, respectively. The 18 h bacterial cultures in CAMHB were adjusted to 0.85% NaCl to achieve 10^8^ cfu/ml as previously described and then diluted to 10^7^ cfu/ml. Finally, the 1 *μ*l containing 10^7^ cfu/ml of each bacterium was separately spotted onto overdried MHA surface with various concentrations of CRE and ampicillin as mentioned above. The final concentration of ARSA per spot was 10^4^ cfu/spot. All dilutions and the spots were performed in triplicate. Then all 6-well plates were incubated at 37°C for 18 h. The lowest concentration of CRE and ampicillin with no bacterial growth was defined as MIC.

### 2.8. Checkerboard Assay

The combined effect of CRE plus ampicillin was assessed by a checkerboard assay determination using the agar dilution technique. In addition, the checkerboard and the MIC were performed simultaneously following the method of Fratini et al. (2017) with slight modifications [22]. This was performed in the same manner as a MIC determination except the CRE and ampicillin were combined. The nine final concentrations of CRE and ampicillin in the combination were ranged as 4MIC, 2MIC, MIC, MIC/2, MIC/4, MIC/8, MIC/16, MIC/32, and MIC/64 for each substance. The 0.2 ml of DMSO without an antibacterial agent in 1.8 ml MHA was employed as a negative control. Finally, the 1 *μ*l containing 10^7^ cfu/ml of 18 h ARSA strains was spotted onto overdried MHA surface containing various concentrations of CRE plus ampicillin. All dilutions and the spots were performed in triplicate. Then all 6-well plates were incubated at 37°C for 18 h. The checkerboard assay and the isobologram were plotted: *y*-axis was CRE concentration in mg/ml, while *x*-axis was the ampicillin concentration in *μ*g/ml. Fraction inhibitory concentration (FIC) index of the antibacterial combination was calculated by the following formula to determine synergistic activity [22–24].

FIC index (FICI) = FIC_A_ + FIC_B_ = [A] in MIC of [A + B]/MIC of A alone + [B] in MIC of [A + B]/MIC of B alone. According to Odds (2003), the FICI value of a combination ≤ 0.5 is considered synergistic (Syn_O_); FICI > 0.5–4.0 is considered as no interaction or indifferent (Ind_O_); FICI > 4.0 is considered antagonistic (Ant_O_) [24]. Besides, EUCAST (2000) determines when FICI value ≤ 0.5 is defined as synergistic effect (Syn_E_), additive effect (Add_E_, 0.5 < FICI value ≤ 1.0), indifferent effect (Ind_E_, 1.0 < FICI value < 2.0), and antagonistic effect (Ant_E_, FICI value ≥ 2.0) [23]. Furthermore, according to Fratini et al. (2017), when the FICI of the combination is <1.0, the combination is defined as synergistic (Syn_F_); when FICI is 1.0, it indicates a commutative effect (Com_F_); when 1.0 < FICI value ≤ 2.0, it indicates “an indifferent effect (Ind_F_)” between the agents, and FICI value > 2.0 is termed as an antagonistic effect (Ant_F_) between the two compounds [22] (Table 4).

### 2.9. Time Killing Assay

Time killing assay was employed to evaluate the antibacterial activity of CRE either alone or in combination with ampicillin and confirmed checkerboard assay. The assay was achieved by using the methods previously described [25]. The 5 × 10^6^ cfu/ml of mid-log phase ARSA in 0.85% NaCl was subjected to each 45 ml CAMHB containing sub-MIC (1/2-MIC) of CRE and ampicillin when used singly. The same concentration of ARSA suspension was also inoculated into 45 ml CAMHB containing MICs of the combination of CRE plus ampicillin (0.125 mg/ml of CRE mixing with 1 *μ*g/ml of ampicillin). This made the final concentration of each ARSA per test be 5 × 10^5^ cfu/ml. Furthermore, the 5 ml of 5 × 10^6^ cfu/ml ARSA suspension in 45 ml of CAMHB without antibacterial substance was used as a control. All test samples and control were incubated at 37°C. The 1 ml of each test was taken every 1 h interval, ranging from 0 to 6 h and at 24 h for viable plate counts. The 0.1 ml of each taken sample was dropped and spread on overdried MHA. All viable plate counts were performed in triplicate. Time killing assay curve was plotted: *y*-axis was bacterial cfu/ml, and *x*-axis was a time in an hour. Viable cell count decrease ≥2log_10_ cfu/ml compared to the most active single agent after 24 h of incubation was determined as synergistic activity. Moreover, bactericidal effect was defined as a ≥3log_10_ cfu/ml decrease compared with the initial inoculum after 24 h of incubation [26].

### 2.10. Cytoplasmic Membrane (CM) Permeability Assay

The alteration in the cytoplasmic membrane (CM) permeability can be determined by the leakage of materials absorbing at 260 nm (OD), mostly DNA and RNA. The methods of Siriwong et al. and Shen et al. were followed [25, 27]. The 5 × 10^6^ cfu/ml of mid-log phase ARSA in 0.85% NaCl was subjected to every 180 ml of 2.5 mM sodium HEPES buffer pH 7.0 with 100 mM glucose containing sub-MIC (1/2-MIC) of CRE and ampicillin when used singly. The ARSA suspension was also inoculated into 180 ml of the same buffer containing half of the MICs of the combination of CRE plus ampicillin (0.0625 mg/ml of CRE mixing with 0.5 *μ*g/ml of ampicillin). This made the final concentration of each ARSA per test be 5 × 10^5^ cfu/ml. In addition, the 20 ml of 5 × 10^6^ cfu/ml ARSA suspension in 180 ml of this buffer without antibacterial substance was used as a negative control while the ARSA suspension in the buffer containing nisin (8 *μ*g/ml as final concentration) was used as a positive control. All test samples and controls were incubated at 37°C. The 5 ml of each test was taken every 1 h interval, ranging from 0 to 6 h. The taken samples of each interval were immediately filtered through sterile cellulose acetate membrane filter 0.22 *μ*m. The 200 *μ*l of each filtrate was transferred to 96-well microplates for UV visible range and then the O.D. was measured at 260 nm. The 200 *μ*l pretreatment filtrate (before adding ARSA suspension) of each test was used as a blank for its corresponding test sample. All O.D. measurements were performed in triplicate. Cytoplasmic membrane (CM) permeability curve was plotted: *y*-axis was O.D. at 260 nm and *x*-axis was a time of hours [25, 27].

### 2.11. Transmission Electron Microscopy (TEM)

TEM preparations were carried out in accordance with previously reported and slight modifications [25, 28]. This was performed in the same manner at a time killing assay. The 10^8^ cfu/ml of mid-log phase ARSA in 0.85% NaCl was subjected to each 199 ml CAMHB containing sub-MIC (1/2-MIC) of CRE and ampicillin when used singly. The same concentration of ARSA suspension was also inoculated into 199 ml CAMHB containing half of the MICs of the combination of CRE plus ampicillin (0.0625 mg/ml of CRE mixing with 0.5 *μ*g/ml of ampicillin). This made the final concentration of each ARSA per test be 5 × 10^5^ cfu/ml. The 1 ml of 10^8^ cfu/ml ARSA suspension in 199 ml of CAMHB without antibacterial substance was used as a control. All test samples and control were prepared in triplicate and then incubated at 37°C for 4 h. Each sample was harvested and washed with 0.1 M phosphate buffer pH 7.2. Then the pellets were fixed with 2.5% glutaraldehyde in 0.1 M phosphate buffer pH 7.2 and incubated overnight at 4°C. After washing with 0.1 M phosphate buffer pH 7.2, the 1% osmium tetroxide in 0.1 M phosphate buffer pH 7.2 was added to each sample, mixed, and washed with distilled water. A graded series of increasing acetone concentrations were employed to dehydrate the bacterial cells. The 20%, 40%, 60%, 80%, and 100% acetone were subsequently added to each sample and then centrifuged at 4°C for 5 min to remove the supernatant. A series of acetone: resin was applied to infiltrate the bacterial cells. The acetone : resin (2 : 1 and 1 : 1) was subsequently added to each bacterium sample and incubated at room temperature for 3 and 12 h, respectively. After removing the supernatant, the pure resin was added to each sample and incubated at room temperature for 3 h followed by gently removing the upper part of the resin. By embedding, the pure resin was additionally added to each sample and stood to allow the upper and lower parts of the resin to become homogeneous for 2 h and then incubated at 70°C for 8 h. Each embedding sample was removed from the Eppendorf tube followed by sectioning using a diamond knife on an ultramicrotome. The samples were transferred onto the grids in triplicate and then stained with uranyl acetate and lead citrate in sequence followed by washing with boiled distilled water (absence of CO_2_). Finally, all specimens were examined with a transmission electron microscope JEM-1230 (JEOL). Furthermore, the bacterial cell area of each treatment in electron micrographs was calculated from the bacterial cell width (nm) × bacterial cell length (nm) to assert the effect of CRE either used singly or in combination with ampicillin on ARSA cell size.

### 2.12. *β*-Lactamase Enzyme Assay For *β*-Lactamase Enzyme Inhibitor

The *β*-lactamase enzyme assay was applied to test for the presence of a substance which can act as an inhibitor to inactivate the *β*-lactamase enzyme. The ability of CRE to deteriorate *β*-lactamase type IV activity of* Enterobacter cloacae* was performed following the method as previously described [25, 28]. Briefly, the enzyme activity of *β*-lactamase was adjusted to the sufficient concentration to hydrolyze 50–60% of the substrate, benzylpenicillin (penicillin G sodium salt), within 5 min. The 10 mM ammonium acetate (pH 4.5 acetic acid) : acetonitrile (75 : 25) was used as a mobile phase. Each sample stock of ampicillin in distilled water, CRE in DMSO, and the combination of CRE plus ampicillin including a control (mobile phase without any antibacterial agent) was preincubated with *β*-lactamase enzyme stock solution for 5 min at 37°C before adding benzylpenicillin stock solution followed by incubation at 37°C for 20 min. The final concentrations of ampicillin, CRE, and the combination of CRE plus ampicillin in the test were as follows: 32 *μ*g/ml (1/2-MIC), 0.25 mg/ml (1/2-MIC), and 0.0625 mg/ml of CRE plus 0.5 *μ*g/ml of ampicillin (1/2-MICs of the combination). The final concentrations of *β*-lactamase enzyme and benzylpenicillin in the test were 1250 *μ*g/ml and 100 *μ*g/ml, respectively. For every 5 min interval (ranging from 0 to 20 min), the enzyme activity in the test sample was immediately stopped by the stopping agent, methanol : acetic acid (100 : 1). Finally, the 20 *μ*l of hydrolyzed benzylpenicillin was subjected to high performance liquid chromatography (HPLC) for measuring the remaining substrate. The HPLC was operated with the following setting: injection volume 20 *μ*l, column C18 (5 *μ*m, 250 × 4.6 mm) (Bio-sil), column temperature 35°C, flow rate 1 ml/min, UV detector at 200 nm, and run time of 5 min. The remaining benzylpenicillin substrates were calculated by comparing the peak area of the chromatographic curve and a predetermined benzylpenicillin standard curve.

### 2.13. Statistical Analysis

CM permeability assay, *β*-lactamase enzyme assay, and TEM were carried out in triplicate. The data were expressed as a mean ± standard error of the mean (SEM). Significant differences between these groups were examined using one-way ANOVA and Tukey's HSD post hoc test; *p* < 0.01 were considered to be a significant statistical difference [25, 28, 29].

## 3. Results

### 3.1. Percent Yield, Qualitative, and Quantitative Phytochemical Screening

The percentage yield of CRE was 11.34% (w/w) of the mass of dry crude (Table 1). The phytochemical compounds of CRE were positive for alkaloids, flavonoids, tannin, and polyphenols but negative for saponin and glycosides. The results indicated that the TAC, TFC, and TPC of CRE were 0.0000703 mg of RE/g, 0.077825 mg of QE/g, and 0.110772 mg of GAE/g dry weight of CRE, respectively (Table 1).

### 3.2. Agar Disc Diffusion Screening

CRE at 250 mg/ml gave the highest inhibition zone for both* S. aureus* sensitive strain and all* S. aureus* DMST strains at 12 mm and 15 mm, respectively (Table 2). Furthermore, 10 mm of the ampicillin inhibition zone was shown for* S. aureus* sensitive strain, whereas there was no inhibition zone for all* S. aureus* DMST strains treated with ampicillin (Table 2), even though the inhibition zone of all* S. aureus* DMST strains treated with CRE contributed wider diameter (15 mm) comparing to that of* S. aureus* sensitive strain (12 mm).

### 3.3. Minimum Inhibitory Concentration (MIC) Determination and Checkerboard Assay

All* S. aureus* DMST strains treated with CRE displayed the MIC result at 0.5 mg/ml which was higher than that of* S. aureus* sensitive strain at 0.25 mg/ml (Table 3). The bacteria treated with ampicillin also showed the MIC results at 0.25 *μ*g/ml for sensitive strain and 64 *μ*g/ml for all* S. aureus* DMST strains (Table 3). According to the results, checkerboard assay with the lowest FICI at 0.27 indicated synergistic activity of the combination of CRE (0.125 mg/ml) plus ampicillin (1 *μ*g/ml) against all* S. aureus* DMST strains as shown in Table 3. The concentration of ampicillin that can inhibit all* S. aureus* DMST strains growth had considerably reduced from 64 *μ*g/ml to 1 *μ*g/ml in combination with CRE. The FICI values ranging from 0.27 to 2.0 were obtained. The highest FICI value of 2.0 was detected in 3 strains. The FICI interpretation displayed that the FICI frequency of Syn_O_, Syn_E_, and Syn_F_ was 13.85%, 13.85%, and 29.23%, respectively (Table 4). The highest FICI frequency was observed in Ind_O_, Ind_F_, and Ind_E_ of 86.15%, 69.23%, and 55.38% respectively, whereas, Ant_O_, Ant_E_, and Ant_F_ were not appearing.

### 3.4. Time Killing Assay

The survival rates of ARSA with various treatments such as ampicillin and CRE, when used singly, including the combination of CRE plus ampicillin were determined by time killing assay.

**Figure 1:**
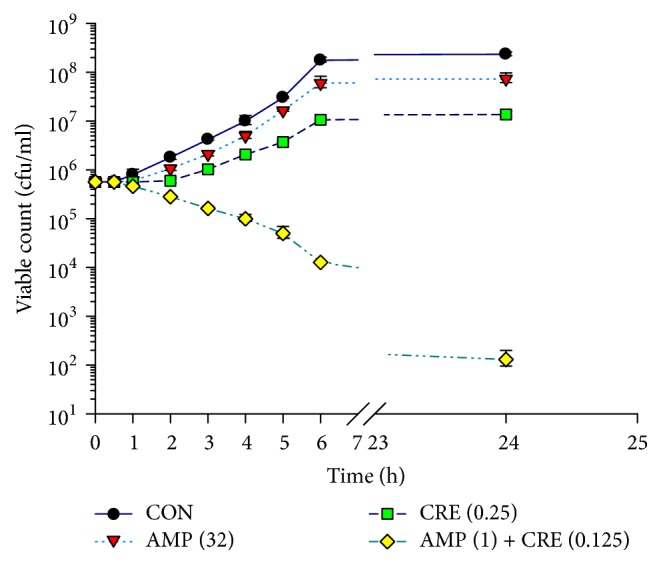
Time killing curve of ARSA after exposure to CRE and ampicillin either alone or in combination. CON = control (drug free); CRE (0.25) = CRE at 0.25 mg/ml; AMP (32) = ampicillin at 32 *μ*g/ml; AMP (1) + CRE (0.125) = ampicillin at 1 *μ*g/ml plus CRE at 0.125 mg/ml; the values plotted are the means of 4 observations, and the vertical bars indicate the standard errors of the means.

Treatment with 0.85% NaCl, a negative control, had no effects on viability. Similarly, individual treatment with CRE or ampicillin slightly decreased viability. However, we found that combination treatment with CRE and ampicillin remarkably decreased cell viability by 6 h, and reduced viability (to around 5 × 10^2^ cfu/ml) was sustained until the end of the experiment (24 h). Our findings reflected those of the checkerboard assay, which demonstrated synergism of combination treatments, illustrated by decreased cell numbers (≥2log_10_ cfu/ml), in comparison with cells treated with the only CRE at 24 h. In addition, the CRE and ampicillin combination exhibited bactericidal effect by a reduction in >3log_10_ cfu/ml in comparison to the initial inoculum at 24 h [26] (Figure 1).

### 3.5. Cytoplasmic Membrane (CM) Permeability Assay

**Figure 2:**
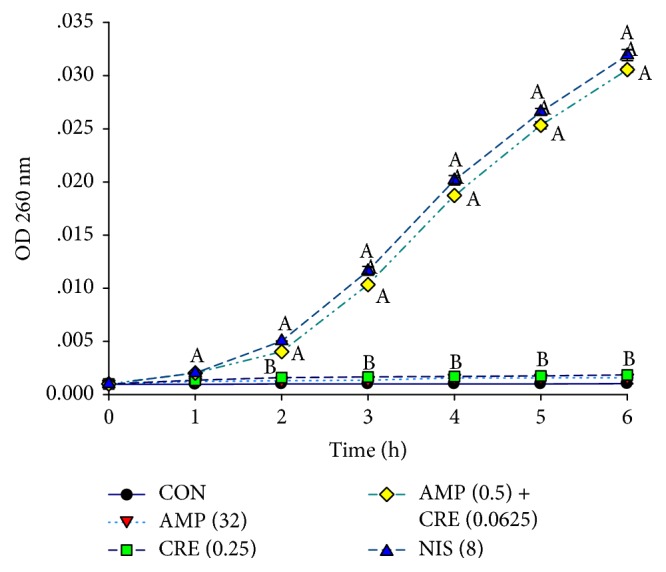
ARSA CM permeability measurements over time following exposure to ampicillin and CRE either alone or in combination. CON = control (drug-free); AMP (32) = ampicillin at 32 *μ*g/ml; CRE (0.25) = CRE at 0.25 mg/ml; AMP (0.5) + CRE (0.0625) = ampicillin at 0.5 *μ*g/ml plus CRE at 0.0625 mg/ml; NIS (8) = nisin concentration at 8 *μ*g/ml. The values plotted are the means of 4 observations, and the vertical bars indicate the standard errors of the means. Different letters indicate groups with statistical significance compared with other groups (Tukey's HSD test, *p* < 0.01).

The determination of the alteration in the cytoplasmic membrane (CM) permeability was one of the methods employing to investigate mechanisms of actions of CRE alone and in combination with ampicillin to discover how these antibacterial agents effects on ARSA. The alteration in CM permeability of ARSA treated with ampicillin or CRE, alone, was not significantly different from those of each other including control (ARSA without an antibacterial agent) as shown in Figure 2. In contrast, the CM permeability of ARSA treated with the combination of CRE plus ampicillin was significantly different from that of control and the others (*p* < 0.01) but not significantly different from that of nisin (*p* > 0.01). These data indicate that CRE in combination with ampicillin can increase CM permeability of ARSA. Furthermore, the results of the killing curve and CM permeability provide evidence that the synergistic activity between the CRE and ampicillin against ARSA is enhanced in the log phase, approximately 2–6 h (Figures 1 and 2).

### 3.6. Transmission Electron Microscopy (TEM)

**Figure 3:**
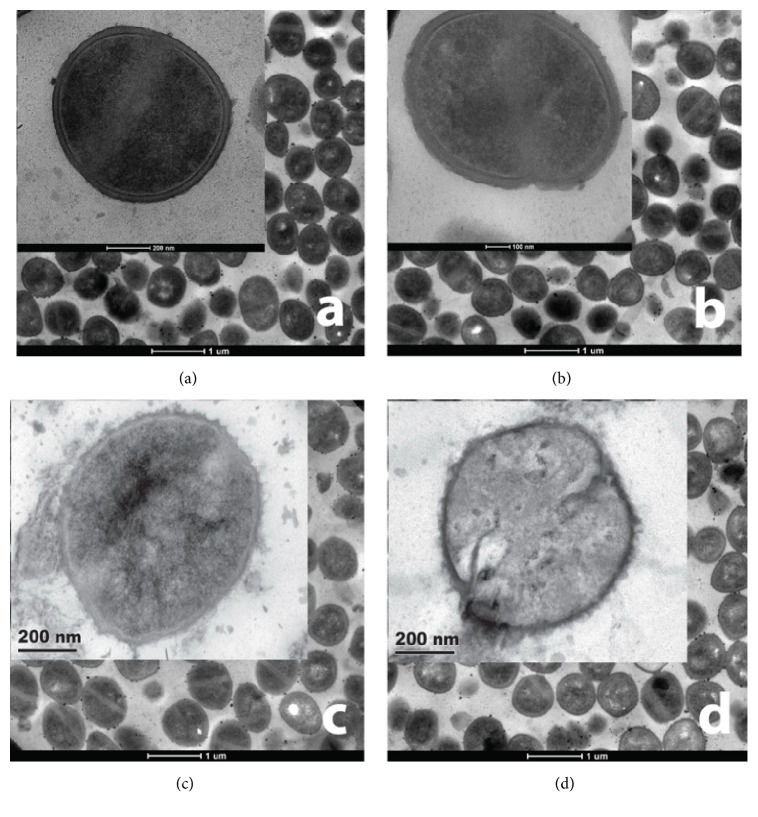
Ultrathin sections of log phase of ARSA DMST 20651 grown in CAMHB: Control (a) bar = 1 *μ*m, ×7000;* inset*: bar = 200 nm, ×38000); 32 *μ*g/ml ampicillin (b) bar = 1 *μ*m, ×7000;* inset*: bar = 100 nm, ×43000); 0.25 mg/ml CRE (c) bar = 1 *μ*m, ×7000;* inset*: bar = 200 nm, ×38000); 0.0625 mg/ml CRE plus 0.5 *μ*g/ml ampicillin (d) bar = 1 *μ*m, ×7000;* inset*: bar = 200 nm, ×38000).

**Figure 4:**
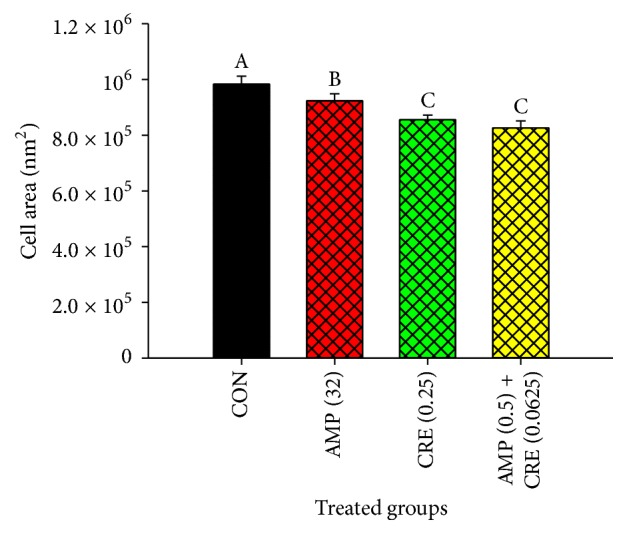
The cell area of ARSA after treatment with CRE and ampicillin either alone or in combination. CON = control (drug-free); AMP (32) = ampicillin at 32 *μ*g/ml; CRE (0.25) = CRE at 0.25 mg/ml; AMP (0.5) + CRE (0.0625) = ampicillin at 0.5 *μ*g/ml plus CRE at 0.0625 mg/ml. The graph shows an area of cell determined by cell width × cell length (nm^2^). The different superscript alphabets are significantly different from each other. Each treated group was compared using one-way ANOVA and Tukey's HSD post hoc test; *p* < 0.01 are presented.

Transmission electron microscopy was manipulated to view the damage of the cell wall of the ARSA treated by either CRE or the combination between the CRE and ampicillin. The electron micrographs for ARSA treated with ampicillin alone exhibited slight damage of the cell wall (30–40%) while the ARSA treated with singly CRE exhibited moderate damage of the cell wall (60–70%), comparing to that of control (Figure 3). In contrast, the most virulent cell wall damage (80–90%) was illustrated in the treatment of ARSA with the combination of CRE plus ampicillin at the mid-log phase, 4 h (Figure 3). In addition, the cell size of ARSA without antibacterial agent, control, (983166.67 ± 9575.72 nm^2^) seemed to be larger than those of the other treatments including ampicillin (923466.67 ± 8490.26 nm^2^), CRE alone (855453.33 ± 5635.96 nm^2^), and the combination of CRE plus ampicillin (826106.67 ± 8414.52 nm^2^) as shown in Figure 4. The results from the cell size study provide evidence that the cell area of ARSA treated with the combination of CRE plus ampicillin exhibit significantly less than those of controls (ARSA without an antibacterial agent) (*p* < 0.01) (Figure 4).

### 3.7. *β*-Lactamase Enzyme Assay For *β*-Lactamase Enzyme Inhibitor

**Figure 5:**
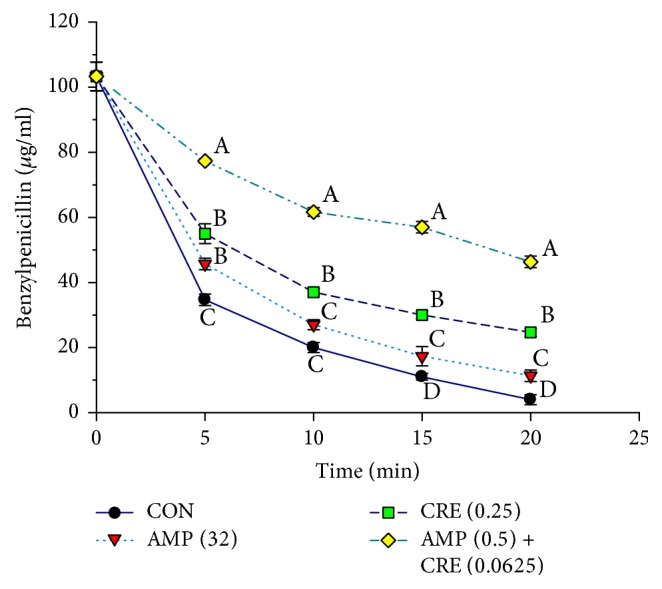
The inhibitory activity of SSE against *β*-lactamase type IV from* E. cloacae* in hydrolyzing benzylpenicillin; CON = control (no testing agent); AMP (32) = ampicillin at 32 *μ*g/ml; CRE (0.25) = CRE at 0.25 mg/ml; AMP (0.5) + CRE (0.0625) = ampicillin at 0.5 *μ*g/ml plus CRE at 0.0625 mg/ml. The graph shows the remaining benzylpenicillin at the same time. Means sharing the same superscript are not significantly different from each other (Tukey's HSD, *p* < 0.01).

The *β*-lactamase enzyme assay was employed to detect the presence of *β*-lactamase inhibitor. The enzyme activity of *β*-lactamase was adjusted to the sufficient concentration to hydrolyze 50–60% of the substrate, benzylpenicillin, within 5 min. The remaining benzylpenicillin substrates were calculated from the standard curve (data not shown). The more the *β*-lactamase inhibitor is present, the more the substrate remains. The assay demonstrated that the benzylpenicillin treated with a *β*-lactamase enzyme with the combination of CRE plus ampicillin contributed the most benzylpenicillin remaining which was significantly different from that of control (ARSA without an antibacterial agent) (*p* < 0.01) (Figure 5). This study provides evidence that the chemical compounds contained in the CRE can inhibit *β*-lactamase activity.

## 4. Discussion 

According to the results, the yield of ethanolic CRE gave the percent yield of 11.34% w/w which was not significantly different from the other reports [4, 30]. Alkaloids, flavonoids, tannins, and polyphenols were present in* C. rotundus* crude extract in this study, which was consistent with several studies [4–8]. There were many pieces of evidence revealing antibacterial activities of alkaloids, including sesquiterpene alkaloids, flavonoids, and polyphenols against several Gram-positive and Gram-negative bacteria, and MRSA [31–33]. Therefore, the presence of these bioactive compounds in CRE indicates that this extract might contain antibacterial activity, either for antibiotic-sensitive bacteria or for resistant bacteria. The presence of various types of these compounds may be in charge of the higher antibacterial activity of CRE since they may work together as a natural combination of various bioactive compounds rather than as a single compound, to inhibit bacteria. These results are consistent with some studies showing that the crude extracts from medicinal plants are much more effective than the only pure, active compound, alone [34–36]. Interestingly, the presence of polyphenols in CRE implies that this medicinal plant could be a potential source of natural antioxidant, as well.

Agar disc diffusion results obviously showed that the CRE could inhibit both* S. aureus* sensitive strain and all DMST strains. According to the results, MIC of CRE against the sensitive strain (0.25 mg/ml) was not significantly different from the other reports [37, 38]. The MIC results confirm the agar disc diffusion test that CRE can act as an antibacterial agent for all* S. aureus* test strains. Checkerboard assay with the lowest FICI at 0.27 indicated that the CRE was able to contribute the synergistic activity with ampicillin antibiotic to inhibit ARSA. One approach to treat drug-resistant bacteria is the combination using two or more antibacterial agents [39]. The advantages of this approach are (1) decrease of the emergence of resistant strains due to the simultaneous use of two or more antibacterial agents against bacteria which develop resistance to each antibacterial agent by each different mechanism; the possibility that the bacterial colonies will emerge resistant to all of the antibacterial agents employed is very low; (2) decrease in dose-related toxicity because of reduced dosage; and (3) use for treating polybacterial infections [40]. In a checkerboard assay, uncertainty in the MIC values can significantly affect FICI. The measurement of MIC simultaneously with FIC using the same checkerboard and the same dilutions (of drugs and microorganism) can reduce this error due to day-to-day variations [22, 41]. Therefore, we carried out the range of MICs from 4MIC to MIC/64 for both CRE and ampicillin on this same checkerboard to confirm the certainty of MIC value previously obtained. These were done in triplicate. Based on the interpretation of Odds (2003), EUCAST (2000), and Fratini et al. (2017), the lowest FICI at 0.27 fell into the category of synergistic effect for all models described [22–24]. The synergistic activity of CRE plus ampicillin combination from the checkerboard assay determination could be confirmed by time killing assay (viable cell count decrease ≥2log_10_ cfu/ml comparable to CRE treated alone at 24 h) [26]. Our time killing assay revealed the viable cell count of the CRE-ampicillin treated ARSA at 24 h was dramatically reduced to 1 × 10^2^ cfu/ml which was more than 2log_10_ cfu/ml compared to its most active single agent (CRE at 1 × 10^7^ cfu/ml).

Some publications reported the combinations of antibacterial agents and antibiotics causing an alteration in CM permeability of some Gram-positive and Gram-negative bacteria [42, 43]. The CM permeability indicated that CRE in combination with ampicillin was able to increase CM permeability of ARSA since the CRE may exert its effects through one of the several important mechanisms of action leading to cell lysis. These mechanisms of action may include the damage of the ARSA cell wall during cell division in log phase. The increased CM permeability subsequently caused the leakage of intracellular constituents such as *β*-galactosidase, ATP, DNA, and RNA [27, 44] and, finally, lead to cell lysis. Therefore, in the presence of CRE in combination with ampicillin, the ARSA cell could not maintain its cell shape leading to disruption of the CM permeability and the leakage of essential molecules such as nucleic acid (DNA and RNA) resulting in cell lysis and death. Moreover, the results revealed that the synergistic activity between the CRE and ampicillin contributed highly inhibition for ARSA in the log phase, approximately 2–6 h. According to the electron micrographs, the most virulent cell wall damage (80–90%) was illustrated in the treatment of ARSA with the combination of CRE plus ampicillin in the mid-log phase, 4 h. This result was correlated to the data from both time killing assay and CM permeability which demonstrated that the damage to the ARSA cell originally occurred from the cell wall damage in the log phase, approximately 2–6 h. The *β*-lactamase enzyme assay was applied to test for the presence of a substance which can act as an inhibitor to inactivate the *β*-lactamase enzyme. The results obtained from our study provide evidence that CRE can inhibit *β*-lactamase. Our results are substantial agreement with the study on the inhibition of the *β*-lactamase enzyme by galangin in a concentration-dependent manner [3]. The mechanisms of action of the *β*-lactamase inhibitor in CRE may occur due to the formation of this inhibitor and *β*-lactamase complex leading to the inactivation of *β*-lactamase activity. Thus, the *β*-lactamase enzyme was inactivated by this inhibitor, therefore allowing or enhancing the *β*-lactam antibiotic to damage the cell wall of ARSA [45]. This may be at least one of the major mechanisms of action of CRE-ampicillin combination against ARSA. Therefore, CRE could reverse the resistance of ARSA to the activity of the primary ampicillin antibiotic through *β*-lactamase inhibitor. In fact, the synergistic activity of the combinations of plant crude extracts and antibiotics has been proven by several publications to be an interesting approach to combat antibiotic-resistant bacteria [3, 46].

Moreover, CRE also had the antibacterial effect of its own against antibiotic-resistant bacteria as shown in MIC determination (0.5 mg/ml) similar to the other plant crude extracts reported by some publications [28, 47].

Recent studies demonstrated that *β*-lactamase inhibitors, which possessed *β*-lactam structures caused the induction of *β*-lactamase expression and hyperproduction of *β*-lactamase [1–3]. Therefore, these currently most available *β*-lactamase inhibitors can also lose their activities through the same mechanism as the *β*-lactam antibiotics [3]. So, the other alternative *β*-lactamase inhibitors with non-*β*-lactam structures are urgently needed to overcome the *β*-lactam antibiotic resistance via multiple mechanisms. The CRE contains bioactive compounds such as alkaloids, flavonoids, and polyphenols which may be responsible for ARSA inhibition. The previous study showed that some of the flavonoids, such as galangin, quercetin, and baicalein could act as a *β*-lactamase inhibitor with non-*β*-lactam structure [3]. Thus, the bioactive compounds present in CRE may also act as a non-*β*-lactam *β*-lactamase inhibitor as well.

## 5. Conclusions

Taken all together, CRE not only contained a natural *β*-lactamase inhibitor, which may be either with or without *β*-lactam structure, but also possessed the ability to conquer the *β*-lactam antibiotic-resistant bacteria by other mechanisms which must be further investigated. Therefore, CRE exerts its antiresistant activity through multiple mechanisms to conquer the *β*-lactam antibiotic-resistant bacteria. Because of this, it may be employed for the potential treatment of ARSA, which is almost resistant to practically *β*-lactam antibiotics. Moreover, the oral administration of CRE in rats showed non-acute-toxicity and no mortality or no behavior change for subacute toxicity [48]. This is the first report demonstrating the mechanisms of action of CRE on ARSA. Further investigations should be performed on the active ingredients study, toxicity, and the level of synergistic effect on blood and tissue confirmation in animals and humans.

## Figures and Tables

**Table 1:** Phytochemical screening and total alkaloid, flavonoid, and polyphenol contents of CRE.

Name of the test	Procedure	Results
Alkaloids	Mayer's test	+
	Hager's test	+
Saponins	Froth test	−
Flavonoids	Shinoda's test	+
	Lead acetate test	+
Tannins	Gelatin test	+
Glycosides	Libermann's Test	−
	Keller Killiant's Test	−
Polyphenols	Test for polyphenol	+
Total alkaloid content (mg of RE/g dry weight of CRE)		0.0000703
Total flavonoid content (mg of QE/g dry weight of CRE)		0.077825
Total polyphenol content (mg of GAE/g dry weight of CRE)		0.110772

+ indicates presence; − indicates absence.

**Table 2:** Agar disc diffusion screening of *S. aureus *ATCC 29213 and *S. aureus* DMST strains.

Pathogenic bacteria	Inhibition zone (mm)		
	CRE (250 mg/ml)	AMP (10 *µ*g/disc) (positive control)	10% DMSO (negative control)
^*∗*^ *S. aureus* ATCC 29213	12 mm.	10 mm.	0 mm.
*S. aureus* DMST 20651	15 mm.	0 mm.	0 mm.
*S. aureus* DMST 20652	15 mm.	0 mm.	0 mm.
*S. aureus* DMST 20653	15 mm.	0 mm.	0 mm.

^*∗*^ *S. aureus* ATCC 29213 was used as a reference strain; AMP = ampicillin; CRE = *Cyperus rotundus* extract; DMSO = dimethyl sulfoxide.

**Table 3:** MICs and FICI of CRE and ampicillin when used either alone or in combination against *S. aureus* strains.

Strains		MIC		FIC^*∗∗*^	FICI^*∗∗∗*^
	AMP (g/ml)	CRE (mg/ml)	NIS (g/ml)	AMP+ CRE (g/ml + mg/ml)	
*S. aureus* DMST 20651	64^R^	0.5^ND^	16	1.0 + 0.125	0.27^SI^
*S. aureus* DMST 20652	64^R^	0.5^ND^	16	1.0 + 0.125	0.27^SI^
*S. aureus* DMST 20653	64^R^	0.5^ND^	16	1.0 + 0.125	0.27^SI^
^*∗*^ *S. aureus* ATCC 29213	0.25^S^	0.25^ND^	0.5	NT	NT

^*∗*^ *S. aureus* ATCC 29213 was used as a reference strain; FIC^*∗∗*^ was the lowest FIC value; FICI^*∗∗∗*^ was the lowest FICI value. ^S^Susceptible; ^R^resistant; ^SI^synergistic interaction. ^ND^No data in CLSI; NT = no test; AMP = ampicillin; CRE = *Cyperus rotundus *extract; NIS = nisin.

**Table 4:** FICI interpretation percentages.

FICI classes	Odds		EUCAST		Fratini et al.	
	FICI values	Total (%)	FICI values	Total (%)	FICI values	Total (%)
Syn_O_, Syn_E_, Syn_F_	≤0.5	13.85	≤0.5	13.85	<1.0	29.23
Add_E_	-	-	>0.5–≤1.0	30.77	-	-
Com_F_	-	-	-	-	=1.0	1.54
Ind_O_, Ind_E_, Ind_F_	>0.5–≤4.0	86.15	>1.0–<2.0	55.38	>1.0–≤2.0	69.23
Ant_O_, Ant_E_, Ant_F_	>4.0	0.00	≥2.0	0.00	>2.0	0.00

FICI interpretations as suggested by Odds, EUCAST, and Fratini et al.: Syn_O_, Syn_E_, Syn_F_: synergistic effect by Odds, EUCAST, and Fratini et al., respectively; Add_E_: additive effect by EUCAST; Com_F_: commutative effect by Fratini et al.; Ind_O_, Ind_E_, Ind_F_: indifferent effect by Odds, EUCAST, and Fratini et al., respectively; Ant_O_, Ant_E_, Ant_F_: antagonist effect by Odds, EUCAST, and Fratini et al., respectively.
